# Perception of financial well-being as a factor of physical and mental health of adolescents

**DOI:** 10.1192/j.eurpsy.2021.587

**Published:** 2021-08-13

**Authors:** L. Shaigerova, R. Shilko, O. Almazova

**Affiliations:** Faculty Of Psychology, Lomonosov Moscow State University, Moscow, Russian Federation

**Keywords:** Physical health, adolescents, familial financial situation, mental health

## Abstract

**Introduction:**

The familial financial situation and its perception can be an important factor in the subjective well-being of adolescents, affecting their physical health and psychological state.

**Objectives:**

To identify the correlation between the perception of the familial financial situation, the physical health and various aspects of the psychological state of adolescents were self-assessed.

**Methods:**

The study involved 506 adolescents (217 males and 289 females) aged 14 to 18 years (M=16.46; SD=1.07). We analyzed the relationship between participants’ assessment of their family’s financial situation, its changes over the past three years, and the adolescents’ self-report on their physical health, stress experiences, and feelings of happiness.

**Results:**

Perception of the financial situation (r=0.316;p<0.001) and assessment of its changes (r=0.217;p<0.001) are directly related to the self-assessment of physical health for the entire sample, as well as separately for boys and girls. For the entire sample, there were no links between the perception of the financial situation and the experience of stress and happiness. However, the study of relationships with gender as an independent variable showed that in boys, the financial situation score is associated with feeling happy (r=0.189;p=0.005), and in girls, an inverse relationship was found between the perception of a worsening financial situation and the experience of stress (r=-0.242;p<0.001).

**Conclusions:**

The perception of the financial situation by adolescents affects the self-assessment of physical health by both boys and girls, but affects different aspects of the psychological state, depending on gender. The research was supported by the Russian Science Foundation, with the grant 15-18-00109.

